# Targeting IRAK1 in T-Cell acute lymphoblastic leukemia

**DOI:** 10.18632/oncotarget.4150

**Published:** 2015-06-01

**Authors:** Charles Dussiau, Amélie Trinquand, Ludovic Lhermitte, Mehdi Latiri, Mathieu Simonin, Agata Cieslak, Nawel Bedjaoui, Patrick Villarèse, Els Verhoeyen, Hervé Dombret, Norbert Ifrah, Elizabeth Macintyre, Vahid Asnafi

**Affiliations:** ^1^ Université Paris Descartes Sorbonne Cité, Institut Necker-Enfants Malades (INEM), Institut National de Recherche Médicale (INSERM) U1151, and Laboratory of Onco-Hematology, Assistance Publique-Hôpitaux de Paris (AP-HP), Hôpital Necker-Enfants Malades, Paris, France; ^2^ CIRI, EVIR Team, INSERM, U1111, CNRS, UMR5308, Université de Lyon-1, ENS de Lyon, Lyon, France; ^3^ INSERM, U1065, C3M, Equipe “Contrôle Métabolique des Morts Cellulaires”, Nice, France; ^4^ University Paris 7, Hôpital Saint-Louis, AP-HP, Department of Hematology and Institut Universitaire d'Hématologie, EA, Paris, France; ^5^ PRES LUNAM, CHU Angers Service des Maladies du Sang et INSERM U 892, Angers, France

**Keywords:** T-ALL, IRAK1, kinases, therapeutic target

## Abstract

T-cell acute lymphoblastic leukemia (T-ALL) represents expansion of cells arrested at specific stages of thymic development with the underlying genetic abnormality often determining the stage of maturation arrest. Although their outcome has been improved with current therapy, survival rates remain only around 50% at 5 years and patients may therefore benefit from specific targeted therapy. Interleukin receptor associated kinase 1 (IRAK1) is a ubiquitously expressed serine/threonine kinase that mediates signaling downstream to Toll-like (TLR) and Interleukin-1 Receptors (IL1R). Our data demonstrated that IRAK1 is overexpressed in all subtypes of T-ALL, compared to normal human thymic subpopulations, and is functional in T-ALL cell lines. Genetic knock-down of IRAK1 led to apoptosis, cell cycle disruption, diminished proliferation and reversal of corticosteroid resistance in T-ALL cell lines. However, pharmacological inhibition of IRAK1 using a small molecule inhibitor (IRAK1/4-Inh) only partially reproduced the results of the genetic knock-down. Altogether, our data suggest that IRAK1 is a candidate therapeutic target in T-ALL and highlight the requirement of next generation IRAK1 inhibitors.

## INTRODUCTION

T-cell acute lymphoblastic leukemia (T-ALL) is an aggressive hematological malignancy resulting from the transformation of T-cell progenitors at various stages of development. They represent 10% of pediatric and 25% of adult ALL. Despite improvement in therapeutic protocols that cure nearly 80% children and 50% adults, relapse is common and the prognosis of relapsed T-ALL remains extremely poor [1, 2]. T-ALL oncogenesis results from chromosomal rearrangements, somatic genetic mutations, aberrant oncogene expression and impairment of multiple signaling pathways involving kinases [3–7]. Interestingly, the use of several kinase inhibitors has been shown to be effective in T-ALL [8–10]. Therefore, efficient evaluation of their potential therapeutic impact requires their use in subsets of patients that are most likely to benefit.

Interleukin receptor associated kinase (IRAK1) is a ubiquitously expressed serine/threonine kinase that mediates signal transduction downstream to Toll-like (TLRs) and Interleukin-1 Receptors (IL1R) [11]. Receptor activation results in phosphorylation of IRAK1 on threonine 209 which initiates recruitment of TRAF6, thus activating downstream NF-κB and MAPK pathways [12]. An oncogenic role for IRAK1 has recently been reported in myeloid cancers [13]. IRAK1 transcriptional expression has negative prognostic impact in Myelodysplasic syndromes (MDS) and acute myeloid leukemias (AML). Furthermore *in vivo* targeted inhibition of IRAK1 in a xenograft model of MDS demonstrated survival improvement [13, 14]. Oncogenic activation of the TLR/IL1R pathway is found in several B-cell lymphomas, often in conjunction with the MYD88 L265P gain of function mutation [15] and 100% of primary effusion lymphoma harbor IRAK1 gain of function mutations leading to constitutive IRAK1 activation [16]. An IRAK1/4 inhibitor was also effective in MYD88 L265P mutated diffuse large B cell lymphoma (DLBCL) [17, 18].

We recently investigated the transcriptional expression of receptor and receptor-associated kinases in T-ALL by Taqman low density array (TLDA) [8]. We showed the overexpression of several kinases as compared to their normal thymic counterparts, demonstrating that exploration of the receptor kinome defines a rational strategy for testing kinase inhibition in T-ALL. These data showed that IRAK1 was strongly overexpressed in all categories of T-ALL so we sought to further explore the potential role of IRAK1 as a therapeutic target in T-ALL.

## RESULTS

### IRAK1 is overexpressed and functional in T-ALL

**Figure 1:**
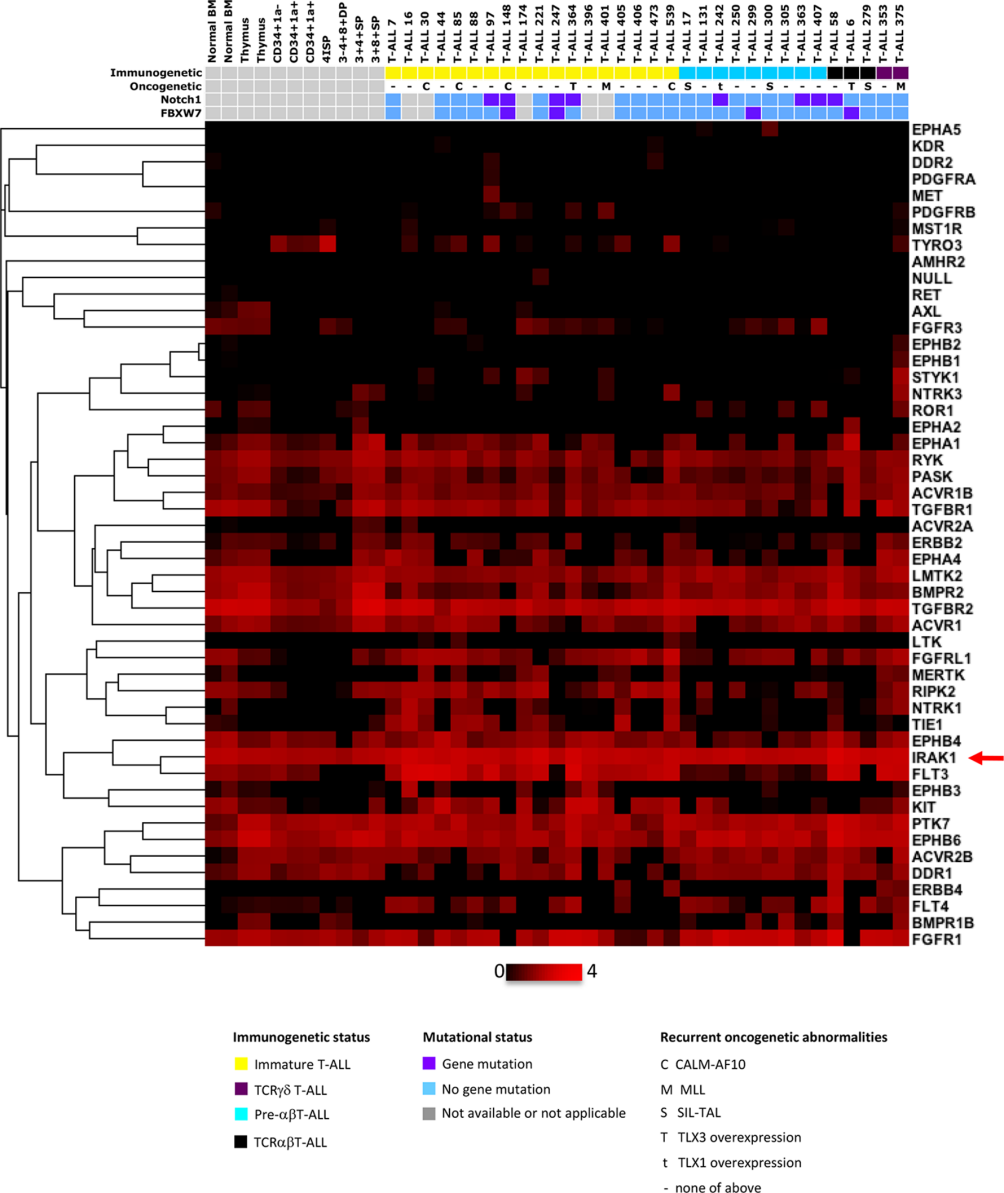
Kinases expression profiles of human T-ALL samples and thymic subpopulations Transcriptional expression of major kinase receptors and receptor associated kinases in normal and malignant immature T-cells. Thymic subpopulations and T-ALL samples are displayed in a supervised classification model and ordered according to their immunogenetic status. Non-expressed (receptor)-kinases are not shown. 4ISP, CD4 immature single positive; DP TCR-, CD4/CD8 double positive surface TCR negative; DP TCR+, CD4/CD8 double positive surface TCR positive; SP4, mature CD4 single positive; SP8, mature CD8 single positive.

**Figure 2:**
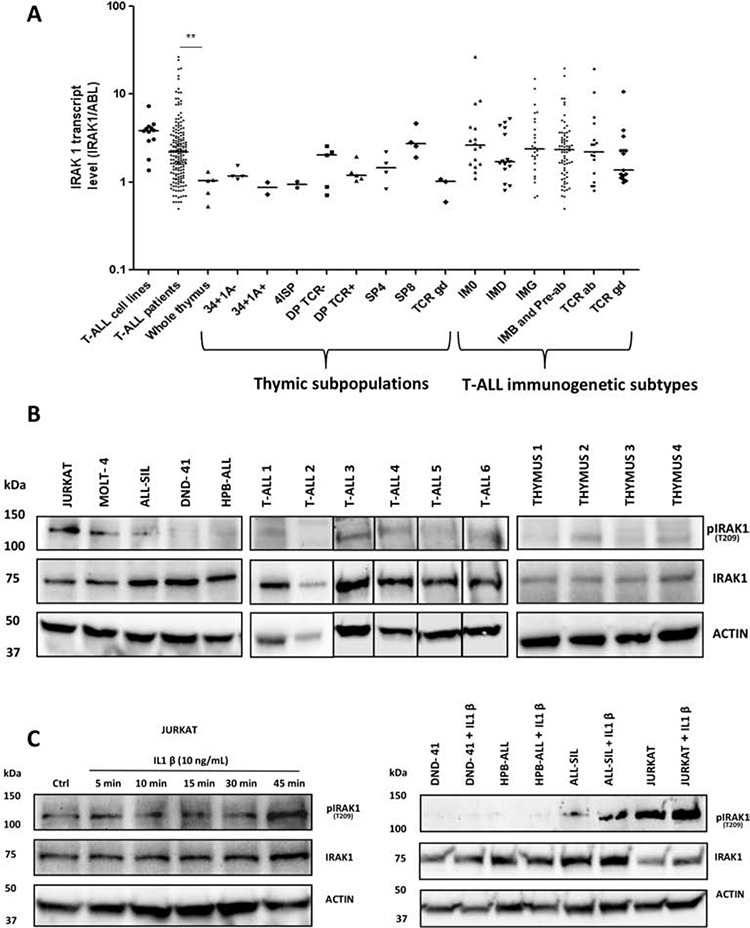
IRAK1 is overexpressed and functional in T-ALL **A.** qRT-PCR: IRAK1 transcriptional expression is shown in T-ALL according to TCR status, and in thymic subsets. **B.** IRAK1 protein expression and phosphorylation were assessed by western blot on T-ALL cell lines, primary T-ALL blasts and normal thymus. **C.** Left panel: Activation of IRAK1 pathway at different time upon IL1β stimulation in the Jurkat cell line. Right panel: Activation of IRAK1 pathway after 45 min treatment withIL1β (10 ng/mL) in T-ALL cell lines. 4ISP, CD4 immature single positive; DP TCR-, CD4/CD8 double positive surface TCR negative; DP TCR+, CD4/CD8 double positive surface TCR positive; SP4, mature CD4 single positive; SP8, mature CD8 single positive; IM0, immature with germline TCR loci; IMB, immature with TCRβ rearrangement; Pre-ab, cTCRβ expressing T-ALL [31].

Transcriptional analysis of the expression level of 65 receptor and receptor-associated kinases in 32 T-ALL (test series) and normal thymic subsets (cell-sorting described in Supplementary Figure S1) showed that IRAK1 was the most highly expressed kinase in all categories of T-ALL, regardless of the immunogenetic stage of arrest or underlying recurrent oncogenetic abnormalities, including Notch1 pathway mutations (Figure 1). We used qPCR to validate the transcriptional pattern of IRAK1 in sorted normal thymic subsets, in T-ALL cell lines, and in a large series of 177 independent (validation series) primary human T-ALL. This confirmed IRAK1 overexpression in T-ALL and cell lines as compared to normal thymus (*p* < 0.01, Figure 2A). IRAK1 transcript levels were slightly higher in most mature TCRab lineage thymic subpopulations as compared to immature and mature TCRgd subsets, without statistical significance (Figure 2A). No difference was observed between mature and immature T-ALL subtypes (Figure 2A) or oncogenic subtypes (not shown) suggesting ubiquitous oncogenic IRAK-1 deregulation in T-ALL, irrespective of stage of maturation arrest and/or oncogenic deregulation.

The IRAK1 protein was also widely expressed in cell lines and primary T-ALL blasts, with a trend to overexpression as compared to normal thymus (Figure 2B). IRAK1 was constitutively phosphorylated on residue T209 at variable levels in lymphoblastic T-cell lines and primary T-ALLs and to a lesser extent in normal human thymus (Figure 2B). In addition, IRAK1 T209-phosphorylation increased over time in the Jurkat T-cell line upon IL-1β stimulation, suggesting a functional IRAK1 pathway (Figure 2C, left panel). Of note, the IL-1β-induced IRAK1 increased phosphorylation was specifically observed in cell lines harboring basal IRAK1 phosphorylation (Jurkat and ALL-SIL T-cell lines) but not in HPB-ALL and DND-41, which express IRAK1 proteins without basal phosphorylation (Figure 2C right panel).

Taken together, these data suggest that IRAK1 is robustly expressed in T-ALL at both transcriptional and protein levels, and remains functional in at least a significant subset of cases.

### Knock-down of IRAK1 induces apoptosis and disrupts cell cycle in T-ALL

**Figure 3:**
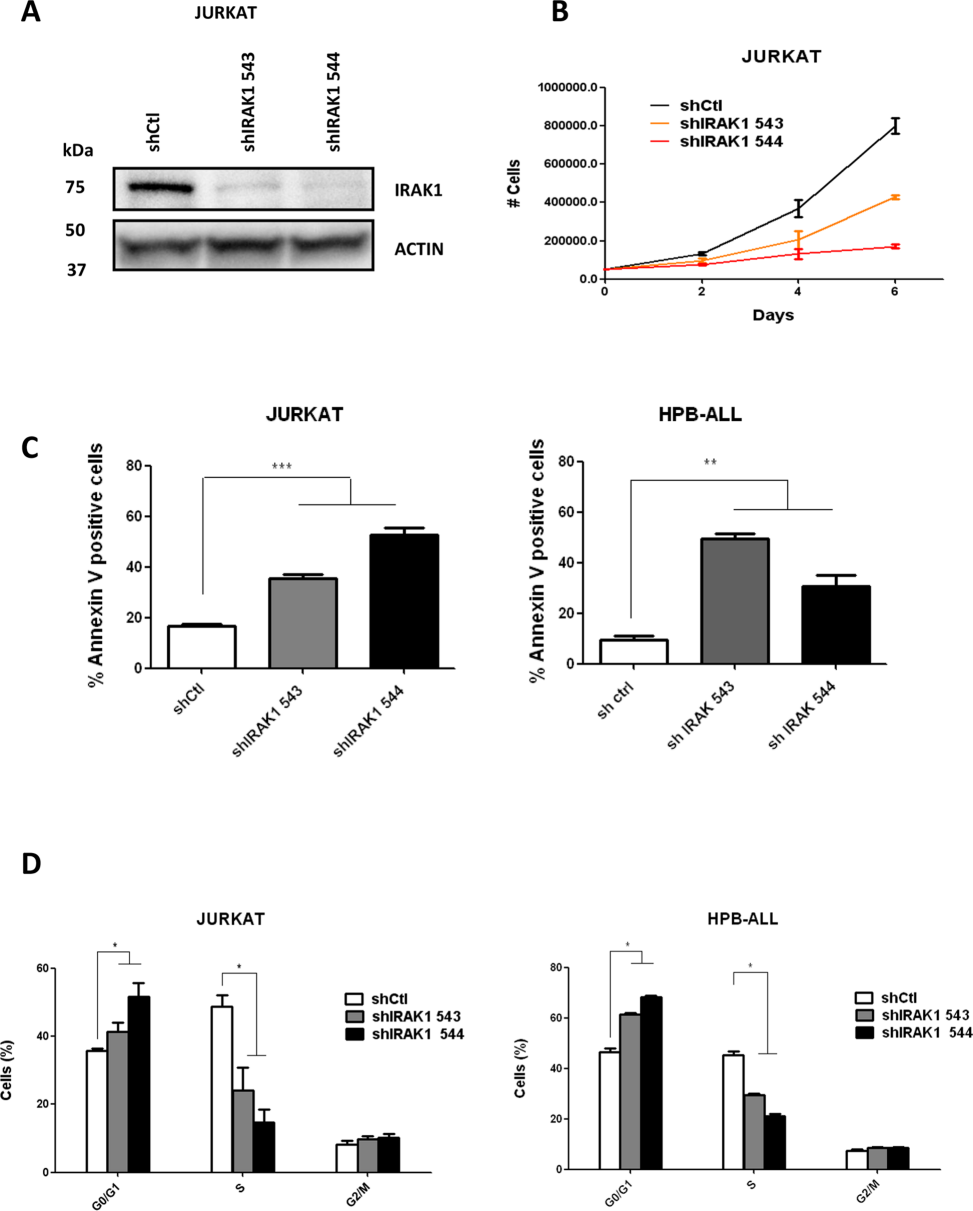
Genetic knock-down of IRAK1 induces apoptosis and disrupts cell cycle **A.** Genetic knockdown of IRAK1 was confirmed by western blotting in Jurkat cells expressing a control or shIRAK1-expressing lentiviral vector **B.** Viable cell growth was assayed by trypan blue exclusion for up to 6 days. Data are represented as mean +/− SEM. **C.** Annexin V/IP staining was assessed in Jurkat and HPB-ALL cells by flow cytometry after transduction with shRNA-expressing lentiviral vectors. **D.** Cell cycle analysis of Jurkat and HPB-ALL cells after transduction was performed with Edu/7-AAD incorporation.

To test whether IRAK1 is required for T-ALL survival, we transduced short hairpin RNAs (shRNA) targeting IRAK1 into T-ALL cell lines. Two independent shRNA (543 and 544) significantly downregulated IRAK1 protein expression in several cell lines (representative data for Jurkat, Figure 3A). Depletion of IRAK1 with both shRNA induced a dramatic decrease in T-cell proliferation (Figure 3B) and increase in apoptosis and cell cycle disruption by increasing the G0/G1 and decreasing the S phase cells in both HPB-ALL and Jurkat cell-lines (Figure 3C and 3D).

### Knock-down of IRAK1 reverses resistance to corticosteroids in Jurkat

**Figure 4:**
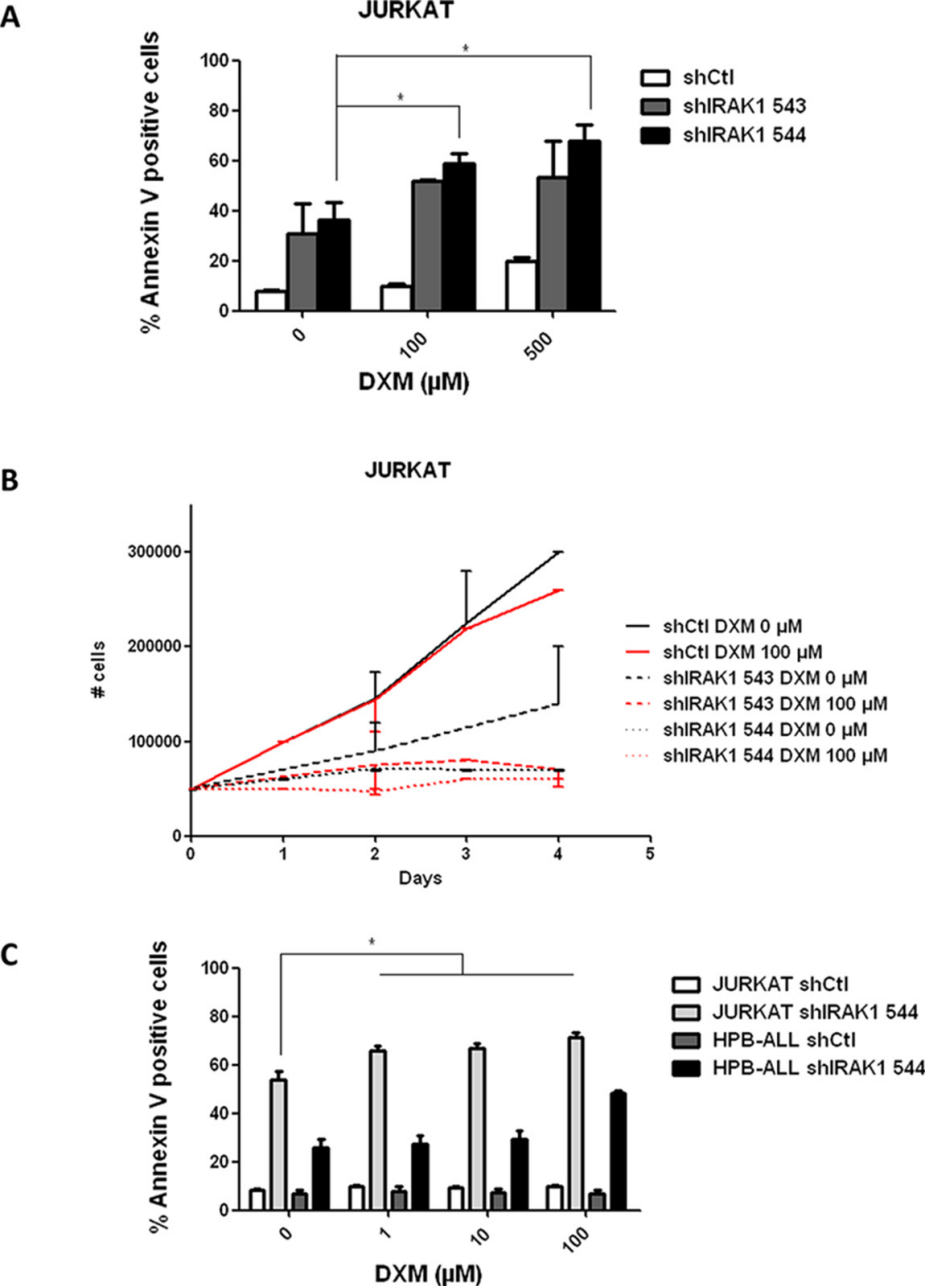
Genetic knockdown of IRAK1 reverses corticoresistance in Jurkat **A.** AnnexinV/IP staining was assessed in Jurkat after transduction with shRNA-expressing lentiviral vector and after 72 h treatment with dexamethasone (0–100-500 μM). **B.** Viable cell growth was assayed by trypan blue exclusion after transduction with shRNA-expressing lentiviral vector and treatment with dexamethasone (0–100 μM) for up to 4 days. Data are represented as mean +/− SEM. **C.** AnnexinV/IP staining was assessed after transduction with shRNA-expressing lentiviral vector in jurkat (white) and HPB-ALL (grey) and after 72 h treatment with dexamethasone (0-1-10-100 μM) on Jurkat (light grey) and HPB-ALL (black) cell-lines. DXM, dexamethasone.

Resistance to corticosteroids remains one of the most challenging issues in ALL therapy. As previously reported [19], the Jurkat cell line is resistant to corticosteroids (Figure 4A). To assess whether knock-down of IRAK1 could reverse this resistance, we tested the effects of dexamethasone in Jurkat after genetic IRAK1 inactivation. Dexamethasone induced apoptosis was significantly increased in cells transduced with IRAK1 shRNA as compared to control cells (Figure 4A). A similar effect was observed on cell proliferation (Figure 4B). In order to confirm these data, we then performed assays with a broader range of dexamethasone doses tested on a p-IRAK positive (Jurkat) and a p-IRAK negative (HPB-ALL) T-ALL cell line with simultaneous genetic knockdown of IRAK-1 using shRNA544 (Figure 4C). These data demonstrated that the corticosteroid response reversion is specifically observed in p-IRAK1 positive cells (Figure 4C). Altogether these data support that knockdown of IRAK1 could sensitize corticosteroid resistant p-IRAK1 positive T-ALL to dexamethasone therapy.

### Pharmacological inhibition only partially reproduces the effects of genetic knock-down

**Figure 5:**
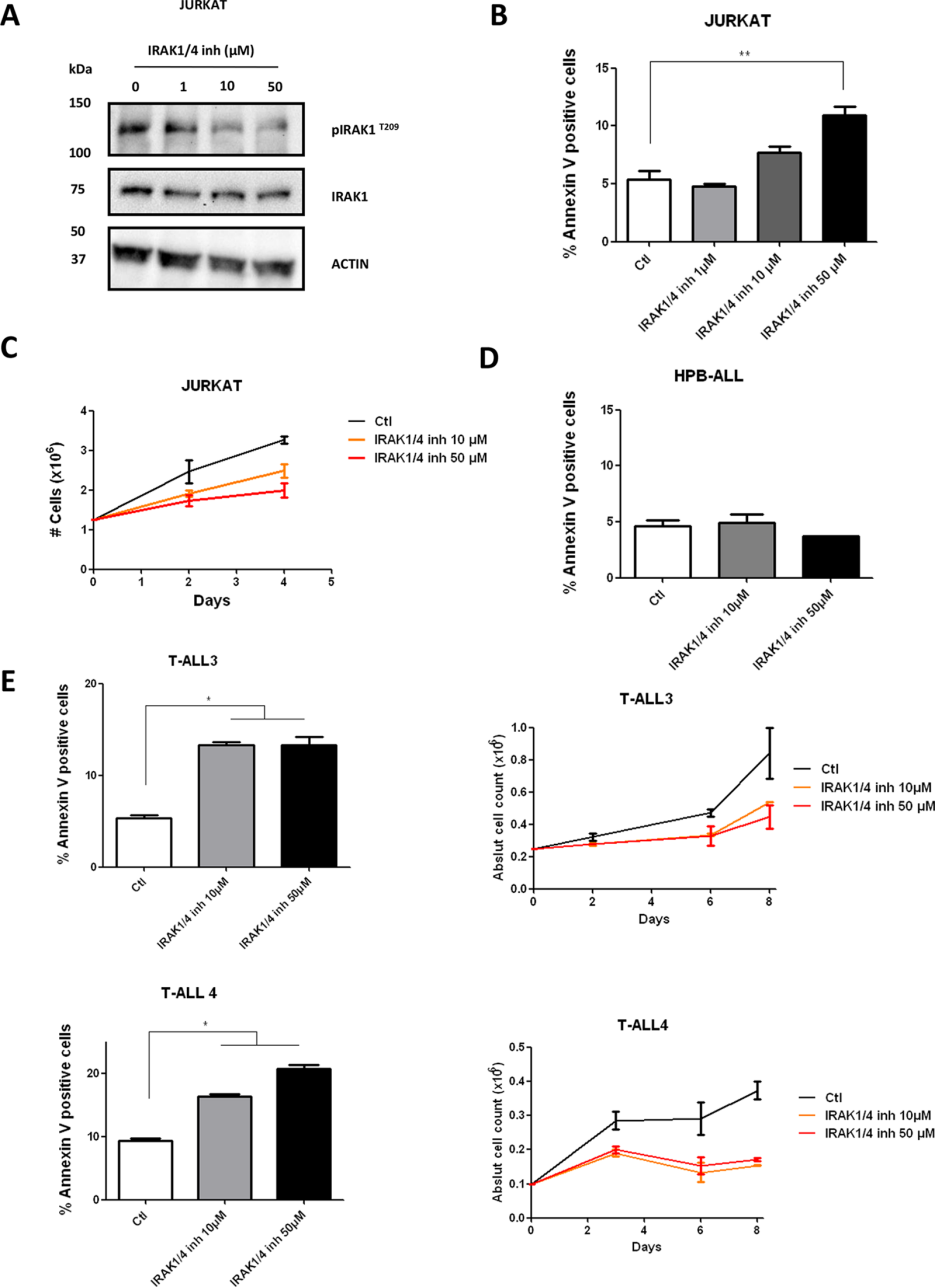
Effect of IRAK1/4-inh on T-ALL cell lines **A.** IRAK1 and pIRAK^T209^ were evaluated by western blotting in Jurkat cells after 48 h treatment with IRAK1/4 inhibitor **B–E.** AnnexinV/IP staining was performed after 72 h treatment with IRAK1/4 inhibitor in Jurkat, HPB-ALL cells and in primary T-ALL blasts from 2 patients (C–E) Viable cell growth of Jurkat and in primary T-ALL blasts from 2 patients was assayed by trypan blue exclusion in the presence of IRAK1/4 inhibitor (0-10-50 μM). Data are represented as mean +/− SEM.

We then submitted Jurkat, to increasing doses of the previously reported pharmacological inhibitor of IRAK1 (IRAK1/4-Inh) [20, 21]. A significant decrease in phosphorylation of IRAK1 was observed after 48 h treatment, demonstrating a biochemical effect of the inhibitor (Figure 5A). However, the apoptotic effect and the decrease in proliferation induced in the Jurkat cell-line upon IRAK1 inhibition were not as dramatic with the pharmacologic inhibition as with the genetic knockdown (Figure 5B and 5C). However, these effects remained significant compared to the HPB-ALL p-IRAK1 negative cell-line (Figure 5D) and are consistent with previously reported data on MDS/AML [13]. To confirm these data, we finally performed pharmacological inhibition of IRAK1 in two primary T-ALL samples (T-ALL3 and T-ALL4). Similarly, we observed a significant increase in cell apoptosis of primary T-ALL samples upon pharmacological inhibition of IRAK1/4 (Figure 5E). Taken together, genetic knock-down and, to a lesser extent, pharmacological inhibition of IRAK1 both resulted in decreased proliferation and induction of apoptosis in the Jurkat cell-line and primary T-ALL blasts.

## DISCUSSION

Improving prognosis remains a therapeutic challenge in T-ALL. Kinases have recently been shown to be druggable targets in T-ALL, particularly in Early T-precursor (ETP)-ALL, a very immature subtype with myeloid features which is associated with a high risk of induction failure or relapse and a poor outcome [22]. Indeed, kinases play a key role in signaling pathways downstream to cytokine receptors that are important in T-ALL and central to ETP ALL oncogenesis [3]. We have now identified IRAK1 as a novel candidate kinase for targeted therapy in T-ALL, including ETP-ALL. IRAK1/4 inhibitor was originally developed for autoimmune and inflammatory diseases [20], and was effective *in-vitro* and *in-vivo* in hematological malignancies (MDS, AML, DLBCL) that overactivate the IRAK1 pathway [13, 18], albeit to a lesser extent. These data in T-ALL underline the requirement of new and more efficient IRAK1 inhibitor for clinical use in leukemia therapeutics.

Our data support that inhibition of IRAK1 activity result in corticoresistance reversion, only in the context of constitutive activation of IRAK1. The unexpected observation that pharmacological inhibition of IRAK1 could not result in comparable cytotoxicity to genetic knock-down of IRAK1 raises the possibility that the cellular oncogenic effects of IRAK1 may not be entirely mediated through its kinase domain. There is precedent for this in alternative disease models, such as the FLT3 receptor which displays both kinase-dependent and independent effects. One mechanism accounting for primary resistance to pharmacological FLT3 inhibition appears to be related to the maintenance of kinase-independent effects of FLT3 upon TKD exposure, with active recruitment of signaling molecules on Grb2 and downstream overexpression of MCL1, thereby maintaining oncogenic FLT3 activity [23]. Interestingly, previous studies showed that kinase-dead IRAK1 mutants maintain IL1-mediated NF-kB and MAPK activation, suggesting that the kinase domain of IRAK1 may be dispensable for the activation of some physiological downstream signaling mediators [24–26]. Whether the present discrepancy between genetic and pharmacological inhibition results from kinase-independent signaling remains to be addressed. However, the exact sequence of molecular events following IRAK1 activation is not fully deciphered and kinase-related and kinase-independent effects remain controversial [27].

We identified deregulation of IRAK1 in all T-ALL subsets but the molecular mechanism(s) of overexpression and constitutive activation in hematological malignancies has not been addressed so far. It may result from post-transcriptional deregulation by miRNA such as miR-146, which targets IRAK1 mRNA [28]. The role of IRAK1 in normal thymopoïesis and in T-ALL pathogenesis should be further investigated. It has been shown that the NF-κB pathway is activated and can be targeted in T-ALL [29]. In this context, targeting IRAK1 may represent a new approach to inhibit the NF-κB pathway preferentially in leukemic compared to normal lymphoid precursors.

In conclusion, our study identifies IRAK1 as a potential novel target in T-ALL, particularly in cortico-resistant T-ALL and ETP-ALL but optimal therapeutic intervention will require development of next generation IRAK1 inhibitors and their preliminary evaluation in *in-vivo* tests and mouse xenograft models of T-ALL.

## MATERIALS AND METHODS

### Patients

Diagnostic peripheral blood or bone marrow samples from 177 adults with T-ALL were analyzed after informed consent was obtained at diagnosis according to the Declaration of Helsinki. Patients were included in the GRAALL03/05 trials, both were registered at ClinicalTrials.gov (GRAALL-2003, NCT00222027; GRAALL-2005, NCT00327678).

Normal thymic samples were obtained from children undergoing cardiac surgery at the Necker-Enfants Malades Hospital, with informed consent from the parents.

### Molecular analysis (Taqman Low Density Array and real-time quantitative PCR)

Quantification of kinase expression was performed on quality-controlled cDNA using predesigned TaqMan Low Density microfluidic cards (TLDA Human Protein Kinase Array; Applied Biosystems). Samples with GAPDH cycle thresholds superior to 21 were excluded from analysis. Each probe value was normalized with respect to GAPDH copy number and analyzed with a Δ13 cycle threshold to discard background noise.

IRAK1 transcript expression was quantified by real-time quantitative PCR in an unselected series of 177 T-ALLs with available cDNA.

### Cell lines and culture conditions

The JURKAT, MOLT-4, ALL-SIL, DND-41, and HPB-ALL T cell lines were grown in RPMI 1640 supplemented with 50 μg/mL streptomycin, 50 IU penicillin, 2 mM L-glutamine and 20% fetal bovine serum (10% for JURKAT). Cell cultures were maintained at 37°C in a humidified atmosphere containing 5% CO_2_. Primary T-ALL were co-cultured on OP9-DL1 in a α-MEM media supplemented with 20% FBS (Hyclone ; Thermo Fisher Scientific), 50 μg/ml streptomycin, 50 IU penicillin and recombinant human cytokines hFLT3-L (5 ng/mL), hIL-7 (2 ng/mL) and hSCF (10 ng/mL) (Miltenyi).

### Reagents

The IRAK1 inhibitor (IRAK1/4 inhibitor or IRAK-Inh; Amgen Inc.) was purchased from Merck Millipore (Ref 407602). Recombinant human IL1-β was purchased from Miltenyi (Ref 130-093-897).

### Apoptosis, cell cycle analysis and growth curves

Apoptosis was assessed by flow cytometry using Annexin V APC (and propidium iodide (BD-Pharmingen, San Jose, CA, USA) staining.

Cell cycle analysis was performed by flow cytometry using EdU/7-AAD staining purchased from Invitrogen, as per manufacturer recommendations.

Cell viability was measured with trypan blue exclusion using a Hemocytometeror an automated cell counter (CASY, Roche Diagnostics).

### Western blotting

T-ALL cell lines and primary T-ALL cells at diagnosis were washed in PBS and lysed in HNTG buffer (50 mM Hepes pH 7.4, 150 mM NaCl, 50mM NaF, 1mM EDTA, 1% Triton, 10% glycerol, 1.5 mM MgCl_2_) supplemented with protease and phosphatase inhibitors (Halt Protease and Phosphatase Inhibitor Cocktail, Thermo scientific). Protein was separated by 7.5% SDS-PAGE (MiniProtean, Biorad) and transferred onto nitrocellulose membranes. After blocking 1 h in TBST 5% BSA, membranes were incubated overnight at 4°C with the first Ab in blocking buffer. Primary Abs used were: anti-pIRAK1 ^T209^ (1/500, Assay Biotech) anti-IRAK1 (1/1000, Cell Signaling) and anti-ACTIN (1/1000, Santa Cruz). After washing with TBST, the membrane was incubated for 30 min at room temperature with the appropriate secondary Ab coupled to HRP in blocking buffer. The signal was detected using the WestDura supersignal or WestFemto supersignal kit (GE Healthcare Bio-Sciences).

### Knock-down of IRAK1 with shRNA lentivirus

Two independent and validated pLKO.1-shIRAK1 constructs developed by Rhyasen et al [13] were used (TRCN0000000543 and TRCN0000000544).

### Statistical analysis

All monoparametric measurement comparisons were determined using the Mann-Whitney test (PRISM software, GraphPad, La Jolla, CA, USA). All Tests were two-sided, with *p* < 0.05 considered statistically significant)

Hierarchical classifications were performed using the Pearson's correlation coefficient as a similarity metric with an average linkage algorithm as described [30].

## SUPPLEMENTARY FIGURE


