# Supplementary material for: Targeting IRAK1 in T-Cell acute lymphoblastic leukemia
Source: Oncotarget. 2015 Jun 1;6(22):18956–65. doi: 10.18632/oncotarget.4150 (PMC4662467; doi:10.18632/oncotarget.4150)
Supplement: Supplementary file 1 [file oncotarget-06-18956-s001.pdf]

SUPPLEMENTARY FIGURE

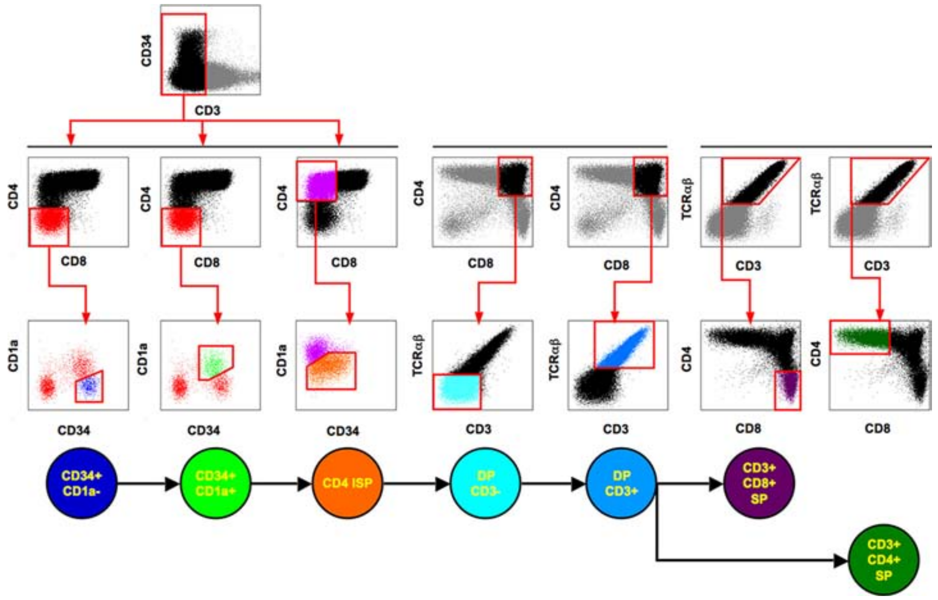

Supplementary Figure S1: Strategy of gate settings used for cell sorting and discrimination of individual stages of T-cell development in human thymus.
